# Automated Real-Time Tumor Pharmacokinetic Profiling in 3D Models: A Novel Approach for Personalized Medicine

**DOI:** 10.3390/pharmaceutics12050413

**Published:** 2020-04-30

**Authors:** Jan F. Joseph, Leonie Gronbach, Jill García-Miller, Leticia M. Cruz, Bernhard Wuest, Ulrich Keilholz, Christian Zoschke, Maria K. Parr

**Affiliations:** 1 Core Facility BioSupraMol, Freie Universität Berlin, 14195 Berlin, Germany; jan.joseph@fu-berlin.de; 2 Institute of Pharmacy (Pharmacology & Toxicology), Freie Universität Berlin, 14195 Berlin, Germany; leonie.gronbach@fu-berlin.de (L.G.); jillg94@zedat.fu-berlin.de (J.G.-M.); leticia.cruz@fu-berlin.de (L.M.C.); christian.zoschke@fu-berlin.de (C.Z.); 3 Agilent Technologies GmbH, 76337 Waldbronn, Germany; bernhard_wuest@agilent.com; 4 Charité–Universitätsmedizin Berlin, corporate member of Freie Universität Berlin, Humboldt-Universität zu Berlin and Berlin Institute of Health, Comprehensive Cancer Center, 10117 Berlin, Germany; Ulrich.Keilholz@charite.de; 5 Freie Universität Berlin, Institute of Pharmacy (Pharmaceutical and Medicinal Chemistry), 14195 Berlin, Germany

**Keywords:** automatization, drug absorption, drug dosing, head-and-neck cancer, pharmacokinetics, real-time measurements, taxanes, tissue engineering, UHPLC-MS/MS

## Abstract

Cancer treatment often lacks individual dose adaptation, contributing to insufficient efficacy and severe side effects. Thus, personalized approaches are highly desired. Although various analytical techniques are established to determine drug levels in preclinical models, they are limited in the automated real-time acquisition of pharmacokinetic profiles. Therefore, an online UHPLC-MS/MS system for quantitation of drug concentrations within 3D tumor oral mucosa models was generated. The integration of sampling ports into the 3D tumor models and their culture inside the autosampler allowed for real-time pharmacokinetic profiling without additional sample preparation. Docetaxel quantitation was validated according to EMA guidelines. The tumor models recapitulated the morphology of head-and-neck cancer and the dose-dependent tumor reduction following docetaxel treatment. The administration of four different docetaxel concentrations resulted in comparable courses of concentration versus time curves for 96 h. In conclusion, this proof-of-concept study demonstrated the feasibility of real-time monitoring of drug levels in 3D tumor models without any sample preparation. The inclusion of patient-derived tumor cells into our models may further optimize the pharmacotherapy of cancer patients by efficiently delivering personalized data of the target tissue.

## 1. Introduction

Selecting clinically relevant doses for the evaluation of anticancer drugs remains challenging in preclinical drug development and contributes to the low translatability of effects in vitro to efficacy in patients. While the understanding of cancer biology advances as the complexity of tumor models and analytical techniques increases, the success rate of drug development in oncology remains the lowest among all therapeutic areas.

Historically, anticancer drug doses for clinical trials have been determined by extrapolating the maximum tolerated dose (MTD) in animals to the human patient. Taking the MTD as the starting point, the effective and safe dose for humans was anticipated in the range of −3 to +3, with three concentrations below and three concentrations above. The revision of this concept is urgently needed, since many nonoptimal doses were taken into late stages of drug development. Especially the testing of high-risk drugs requires a more conservative approach, using the minimum anticipated biological effect level (MABEL) in first-in-human trials [1,2].

Up to now, new concepts focused on the improved extrapolation from animal studies to clinical trials, e.g., by introducing drug metabolism and pharmacokinetic studies in early drug development [3]. In particular, model-based, adaptive/Bayesian approaches already helped to better find effective and safe dosage [4]. Nevertheless, animal models are affected by differences in the human pathophysiology and even xenograft models do not fully recapitulate the barriers of drug uptake into human solid tumors [5].

In fact, drug exposure of tumor cells depends on the architecture of solid tumors with cell density, the spatial arrangement of cells and extracellular matrix proteins, interstitial fluid pressure, and vascular supply [6,7,8]. While 2D monolayer cell culture cannot provide meaningful insights into the pharmacokinetic profiles of solid tumors, sophisticated 3D tumor models such as spheroids or multilayered tumor models could do this, eventually even in a patient-specific manner [9,10].

The introduction of in vitro tumor models into the dose selection for a particular patient requires adapting the protocols to high-content, high-throughput approaches to handle high numbers of tests, e.g., with different drugs and several combinations. However, analytical approaches to quantify drug amounts in tissues comprise imaging- and microdialysis-based methods. While imaging techniques and microdialysis closely map the drug distribution within (tumor) tissues, all methods share the high effort needed in sample preparation [11,12], restricting their use for personalized medicine.

Herein, the development of an in vitro approach for real-time pharmacokinetic investigations in human cell-based models of head-and-neck squamous cell carcinoma is reported. It aims for an automated measurement of docetaxel concentrations within the tumor tissue to quantify the drug absorption. Therefore, an UHPLC-MS/MS method was adapted from clinical practice and optimized for a maximum number of online measurements per time.

## 2. Materials and Methods

### 2.1. Materials

Oral fibroblasts and oral fibroblast medium were purchased from ScienCell (Carlsbad, CA, USA). Tongue cancer cells from the SCC-25 cell line (RRID:CVCL_1682) were a generous gift from Howard Green (Dana-Farber Cancer Institute; Boston, MA, USA) [13]. Collagen G was purchased from Biochrom (Berlin, Germany) and consumables for tumor oral mucosa model culture from Greiner bio-one (Leipzig, Germany). Docetaxel was purchased from Selleckchem (Houston, TX, USA). Acetonitrile, formic acid, methanol, and isopropanol, all LC-MS grade, were purchased from Sigma-Aldrich (München, Germany).

### 2.2. Cell Culture

Oral fibroblasts were precultured in oral fibroblast medium and SCC-25 cells in DMEM/F-12 Ham medium, supplemented with 9% fetal calf serum, 0.9% L-glutamine and 0.9% penicillin/streptomycin at 37 °C and 5% CO_2_. The cancer cells were regularly checked by single nucleotide polymorphism authentication (Multiplexion; Heidelberg, Germany). The medium was changed three times a week and the cells were subcultivated after reaching a confluence of 80%. Cell culture was performed according to standard operating procedures and referred to good cell culture practice.

### 2.3. Sample Port Integration into Tumor Oral Mucosa (TOM) Models

**Figure 1:**
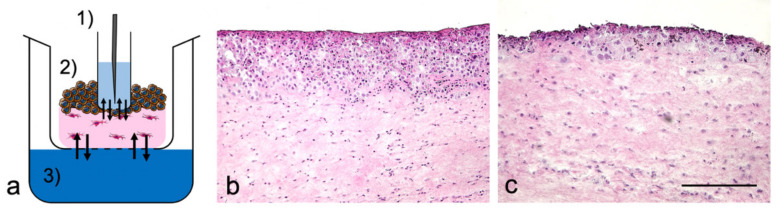
Experimental design and morphology of tumor oral mucosa (TOM) models. (**a**) Schematic cross-section of (1) sampling port with the needle of the autosampler, (2) TOM model with tumor cells (brown) and fibroblasts (magenta) within lamina propria, (3) Reservoir with differentiation medium, supplemented with docetaxel. The arrows indicate drug diffusion equilibria. Hematoxylin and eosin (H&E) staining of TOM models following two applications of (**b**) the vehicle control and (**c**) 7000 ng/mL docetaxel. Images were representative of four batches; scale bar = 250 µm.

Tumor oral mucosa (TOM) models were prepared as described elsewhere [14] but adopted to a 6-well-plate design to handle the integration of the sample port (Figure 1a). In brief, 0.3 × 10^6^ oral fibroblasts were embedded in collagen G and 1 × 10^6^ SCC-25 cells were seeded on top of these lamina propria equivalents one week after. The model growth medium was changed three times a week and replaced by model differentiation medium one week after seeding the tumor cells [14]. The sampling port was created by placing a 24-well insert (400 nm pore size) into the TOM model before the collagen started to solidify. The tumor cells proliferated and migrated into the collagen matrix around the sampling port for seven days, before docetaxel was applied. The 24-well insert was fixed by a custom-made metal support and filled with 600 µL serum-free growth medium. The top of the 6-well plate was sealed with aluminum foil (VWR, Darmstadt, Germany) instead of using the standard plastic lid. TOM models were incubated at 37 °C inside the autosampler of the UHPLC-MS/MS device (Agilent Technologies GmbH, Waldbronn, Germany) for the 96 h observation period in the final week of TOM model culture.

### 2.4. Docetaxel Treatment of TOM Models

Docetaxel was dissolved in DMSO to a 70 mg/mL stock solution and diluted with construct differentiation medium to 7; 70; 700; 7000 ng/mL. DMSO, 0.01% in model differentiation medium, served as vehicle control since this was the maximum DMSO concentration among all samples (0.00001%; 0.0001%; 0.001%; and 0.01% DMSO for 7; 70; 700; 7000 ng/mL docetaxel). Docetaxel solutions were applied two times per construct with an application interval of 48 h.

### 2.5. Morphological Analysis

TOM models were snap frozen at the end of the 96 h observation period and cut into 7 µm thick slices using a cryotome (Leica CM 1510 S; Leica, Wetzlar, Germany). Cryosections were analyzed by hematoxylin and eosin (H&E) staining and pictures were taken with a microscope (BZ-8000; Keyence, Neu-Isenburg, Germany).

### 2.6. UHPLC-MS/MS Analyses

Method A: For automated real-time quantitation of docetaxel, an Agilent 1290 UHPLC coupled to an Agilent 6495 triple quadrupole tandem mass spectrometer equipped with a Jet Stream electrospray ionization (ESI) source was used (Agilent Technologies GmbH, Waldbronn, Germany). Separation of docetaxel was achieved on an Agilent Poroshell Phenyl Hexyl column (50 mm × 2.1 mm, 1.9 µm particle size) equipped with a corresponding guard column (5 mm × 2.1 mm, 1.9 µm particle size) using water (solvent A) and acetonitrile (solvent B) each containing 0.1% formic acid (*v/v*) as mobile phase. At a flow rate of 0.350 mL/min, the following gradient was applied: 5% B for 0.5 min, to 100% B at 4 min, 1 min hold, 5% B at 5.1 min, stop time 6.50 min. The column compartment was kept at 40 °C. The injection volume was 5 µL and the autosampler temperature was set to 37 °C. A needle wash (acetonitrile/methanol/isopropanol/water, 25% each, *v/v/v/v*) was applied for 20 s while an additional needle seat backflush using an Agilent Flex Cube was used to minimize carry over (15 s at 2 mL/min with needle wash solvent, pure isopropanol, and a mixture of water/acetonitrile (95/5, *v/v*) containing 0.1% formic acid). The total run time was 9.75 min.

The mass spectrometer was operated in multiple reaction monitoring (MRM) acquisition mode. Positive electrospray ionization mode (ESI+) yielded the sodium adduct of docetaxel [M + Na]^+^ and was detected at *m/z* 830.3. Source and MRM parameters were optimized using Mass Hunter Source Optimizer software (version 1.1, Agilent Technologies Inc., Santa Clara, CA, USA). Final source parameters were as follows: drying gas temperature: 230 °C, drying gas flow: 20 L/min (nitrogen), nebulizer pressure: 40 psi (nitrogen), sheath gas temperature: 390 °C, sheath gas flow: 12 L/min (nitrogen), capillary voltage: +4,500 V, nozzle voltage: +300 V, high pressure radio frequency (HPRF): 210 V, low pressure radio frequency (LPRF): 160 V. MRM details are listed in Table 1. MassHunter (Quant) software (version B08, Agilent Technologies Inc., Santa Clara, CA, USA) was used for data acquisition and processing.

Method B: For identification of degradation products, an Agilent 1290 II HPLC connected to an Agilent 6550 iFunnel QTOF with Agilent Jet Stream source was used (Agilent Technologies Inc., Santa Clara, CA, USA). Separation of docetaxel and its metabolites was achieved on an Agilent Poroshell Phenyl Hexyl column (50 mm × 2.1 mm, 1.9 µm particle size) equipped with a corresponding guard column (5 mm × 2.1 mm, 1.9 µm particle size) using water (solvent A) and acetonitrile (solvent B) each containing 0.1% formic acid (*v/v*) as mobile phase. At a flow rate of 0.350 mL/min, a longer gradient was applied: 5% B for 0.5 min, to 37% B at 5 min, 50% B at 10 min, to 98% B at 15 min, 2 min hold, back to 5% B at 17.1 min, stop time 19 min. The column compartment was kept at 40 °C. The injection volume was 5 µL. A needle wash (acetonitrile, methanol, isopropanol, water) was applied for 20 s. The mass spectrometric parameters were as follows: drying gas temperature: 230 °C, drying gas flow 14 L/min (nitrogen), nebulizer pressure 40 psi (nitrogen), sheath gas temperature: 375 °C, sheath gas flow: 12 L/min (nitrogen), capillary voltage +4,500 V, nozzle voltage +300 V, high pressure radio frequency 200 V, low pressure radio frequency 100 V, fragmentor 365 V. Data acquisition was performed in auto MS/MS mode using a mass range of *m/z* 100–1000 at a scan rate of 1 spectrum/s for MS1 and *m/z* 50–1000 for MS2 experiments at 3 spectra/s. The collision energy was adjusted depending on the target *m/z* value (offset 4 eV, slope 3 eV/*m/z* 100).

### 2.7. Validation

Method A was used for automated real-time quantitation of docetaxel and validated in terms of selectivity, carry-over, lower limit of quantitation (LLOQ), calibration function, accuracy, and precision following the recommendations of the European Medicines Agency’s (EMA) guideline on bioanalytical method validation [15]. All calibration (CAL) and quality control (QC) samples were freshly prepared in serum-free model differentiation medium as sample diluents.

Selectivity and carry-over: The guidelines require the analysis of matrix from four different lots. Since the matrix was artificial, no remarkable differences had to be considered. Thus, only one batch was used for assessing selectivity. Blank samples (serum-free model differentiation medium) were analyzed and compared with samples spiked with docetaxel at the LLOQ. Less than 20% detector response of the LLOQ is required for the blank samples. Carry-over was determined by analyzing blank samples after the injection of a high concentration QC (HQC) sample (7,500 ng/mL). Again, less than 20% detector response of the LLOQ is required to comply with the EMA guidelines.

Lower limit of quantitation and calibration: The LLOQ needs to be determined with sufficient accuracy and precision and with at least 5 times higher detector response than a blank sample. For evaluation, matrix-matched samples of 0.1, 0.25, 0.5, 1.0, 5.0 ng/mL were investigated. Additionally, the limit of detection (LOD) was determined based on calculations according to ICH guidelines [16]. Calibration samples in medium ranged from the LLOQ of 0.001 µg/mL to the upper limit of quantitation (ULOQ) of 10 µg/mL. In addition to an analyte free matrix sample, eight levels of calibration samples were prepared in triplicate and analyzed on two consecutive days.

Accuracy and precision: Accuracy and precision were assessed on serum-free medium samples spiked with docetaxel at 4 different QC levels with 5 replicates per level in a concentration range from the LLOQ to the ULOQ covering the calibration range. Samples were analyzed on two different days. Mean concentrations and the coefficient of variation (CV) of QC samples were required to be within ±15% in general, or ±20% at the LLOQ of the nominal concentrations, respectively. Within-run and between-run accuracy and precision were determined.

### 2.8. Sample Preparation for the Identification of Degradation Products

The degradation products of docetaxel were analyzed in the differentiation medium cultivated with the models (Table 2). To handle these samples, a protein precipitation procedure was performed. Aliquots of 100 µL of the samples were added to 400 µL of cold acetonitrile and centrifuged at 3328× *g* for 10 min. The serum-free supernatant was then transferred into LC-MS/MS vials for further analysis, according to method B.

### 2.9. Pharmacokinetic Analysis

Pharmacokinetic analyses were conducted in R [17]. First, a non-compartmental analysis was performed. Assumptions were: (i) dose was calculated by concentration in the reservoir x volume (Figure 1a(3)); (ii) area under the concentration curve (AUC) 0–48 h lasted until 48 h and AUC 48–96 h until the end of the experiment; (iii) for the concentration between 48–96 h the unmeasured concentrations were not considered. Afterwards, interval AUCs were calculated. For 0–48 h, the AUC was calculated from 0.0001 h (start of the experiment) to the end of the 1st cycle; for 48–96 h, the AUC was calculated from the time “end of the 1st cycle” to “end of the 2nd cycle”. However, the end of the 2nd cycle varied, since in some experiments, the last concentrations could not be measured. The maximum concentration (C_max_) was determined based on the measured concentrations; time to maximum concentration (t_max_) was the corresponding time to C_max_.

## 3. Results

### 3.1. TOM Models with Sampling Port

The TOM models reproducibly showed an unstructured and hyperproliferative epithelial layer with pleomorphic tumor cells, also separating from the epithelial layer into the lamina propria. Neither the sampling port nor the cultivation within the autosampler of the UHPLC-MS/MS device influenced the tumor growth. The effects of docetaxel on the tumor size in TOM models by supplementing the differentiation medium with either two drug doses or the vehicle control were determined. Whereas the vehicle control did not change the tumor morphology (Figure 1b), docetaxel caused a dose-dependent reduction of tumor size with abundant epithelial cell death (Figure 1c). The average tumor size declined from 347 ± 72 µm (untreated) to 100 ± 45 µm (max docetaxel concentration, *n* = 4 each).

### 3.2. Docetaxel Epimerization and Degradation Products

**Figure 2:**
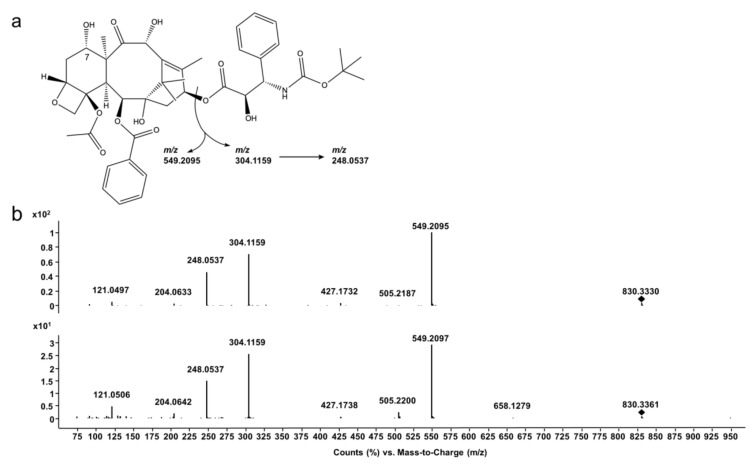
Docetaxel structure fragmentation. (**a**) Chemical structure of docetaxel and main fragmentation products. (**b**) Product ion spectra of docetaxel (top) and its potential 7-epimer (bottom), precursor [M + Na]^+^_theor_ = 830.3358 indicated with black rhombus.

During electrospray ionization, docetaxel mainly forms a sodium adduct ([M + Na]^+^_theor_=830.3358), which is used as precursor ion for all MS/MS experiments. As shown in Figure 2 (top), the product ion spectrum of docetaxel shows three major fragments at *m/z* 549.2095 (taxane nucleus (10-deacetylbaccatin III, 10DABIII), [C_29_H_34_O_9_ + Na]^+^, exact mass *m/z* 549.2095, mass error Δ*m/z* = 0 ppm), *m/z* 304.1159 (phenylpropionic acid side chain, [C_14_H_19_NO+Na]^+^, exact mass *m/z* 304.1155, Δ*m/z* = −1.17 ppm), and *m/z* 248.0537 (side chain with loss of the tert-butyl moiety, [C_10_H_11_NO_5_ + Na]^+^, exact mass *m/z* 248.0529, Δ*m/z* = −3.05 ppm). The two main fragments *m/z* 549. 1 and *m/z* 304.1 were later chosen for MRM transitions in real-time quantitation (method A).

Analyses of docetaxel reference substance as well as cell culture media without cells, and TOM models, revealed a second peak with almost identical MRM transitions (method A, chromatogram showing transitions in Figure S1) and product ions (method B, Figure 2 (bottom)). It already appeared only minutes after preparing the samples for analysis with serum-free medium as sample diluent. This degradation product is postulated to be the 7-epimer of docetaxel (epi-docetaxel), which is known to occur in basic and acidic conditions [18,19]. Since the epimerization could not be avoided in calibration or quality control samples as well, the combined peak areas of docetaxel and the 7-epimer were considered for all further quantitation experiments of docetaxel.

Based on accurate mass data, we postulated further degradation products beside the main degradant epi-docetaxel. Oxidized species of docetaxel and several hydrolysis products (ester and carbamate hydrolysis), as well as oxidation of the products of ester hydrolysis and their respective epimers (Table 2, Figures S3 and S4) are assigned. Two oxidized species of docetaxel show abundant sodium adducts in MS1 of *m/z* 828.3200 (oxo-docetaxel, RT: 9.94 min, [C_43_H_51_NO_14_ + Na^+^]^+^, exact mass *m/z* 828.3202, Δ*m/z* = 0.19 ppm) and *m/z* 828.3192 (epi-oxo-docetaxel, RT: 11.07 min, [C_43_H_51_NO_14_ + Na^+^]^+^, exact mass *m/z* 828.3192, Δ*m/z =* 1.16 ppm). Their MS/MS spectra show abundant fragments at *m/z*_oxo-docetaxel_ 772.2534 and *m/z*_epi-oxo-docetaxel_ 772.2584 ([C_39_H_43_NO_14_ + Na]^+^, exact mass *m/z* 772.2576, Δ*m/z*_oxo-docetaxel_ =−1.07 ppm and Δ*m/z*_epi-oxo-docetaxel_ =5.41 ppm), which may originate from the loss of the tert-butyl residue. They both show a fragment corresponding to an oxidation at the taxane nucleus at *m/z*_oxo-docetaxel_ 547.1927 and *m/z*_epi-oxo-docetaxel_ 547.1955 ([C_29_H_32_O_9_ + Na]^+^, exact mass *m/z* 547.1939, Δ*m/z*_oxo-docetaxel_ = 2.11 ppm and Δ*m/z*_epi-oxo-docetaxel_ = −3.01 ppm). Analogously to docetaxel, the fragment *m/z* 304.1173 originated from the intact phenylpropionic acid side chain ([C_14_H_19_NO_5_ + Na]^+^, exact mass *m/z* 304.1155, Δ*m/z*_oxo-docetaxel_ = −5.77 ppm).

Further degradation products of docetaxel resulted from the ester hydrolysis of the taxane nucleus and the phenylpropionic acid side chain and are postulated here as 10DABIII (*m/z* 545.2378, RT: 4.55 min, [C_29_H_36_O_10_ + H]^+^, exact mass *m/z* 545.2381, Δ*m/z* = 0.60 ppm) and epi-10DABIII (*m/z* 567.2196, RT: 5.31 min, [C_29_H_36_O_10_ + Na]^+^, exact mass *m/z* 567.2201, Δ*m/z* = 0.83 ppm). A loss of benzoic acid, acetic acid and two losses of water from 10DABIII resulted in the fragment *m/z* 327.1587 ([C_20_H_22_O_4_ + H]^+^, exact mass *m/z* 327.1591, Δ*m/z* = 1.18 ppm). Epi-10DABIII showed a fragment at *m/z* 445.1791 ([C_22_H_30_O_8_ + Na]^+^, exact mass *m/z* 445.1833, Δ*m/z* = 9.41 ppm) which may correspond to the loss of the benzoic acid moiety and *m/z* 385.1615 ([C_20_H_26_O_6_ + Na]^+^, exact mass *m/z* 385.1622, Δ*m/z* = 1.71 ppm), which indicates a subsequent loss of acetic acid.

These two hydrolyzed esters most likely exist in an oxidized form as well, which are proposed as oxo-10DABIII (*m/z* 565.2041, RT: 5.40 min, [C_29_H_34_O_10_ + Na]^+^, exact mass *m/z* 565.2044, Δ*m/z* = 0.56 ppm) and epi-oxo-10DABIII (*m/z* 565.2040, RT: 5.84 min, [C_29_H_34_O_10_ + Na]^+^, exact mass *m/z* 565.2044, Δ*m/z* = 0.74 ppm) based on their accurate mass data. They both show a distinct fragment at *m/z*_oxo-10DABIII_ 443.1661 and *m/z*_epi-oxo-10DABIII_ 443.1680 ([C_22_H_28_O_8_ + Na]^+^, exact mass *m/z* 443.1676, Δ*m/z*_oxo-10DABIII_ = 3.47 ppm and Δ*m/z*_epi-oxo-10DABIII_ = −0.81 ppm), most likely originating from the loss of the benzoic acid moiety.

Furthermore, the hydrolysis of the carbamate function of docetaxel revealed two more products: ‘Carbamate’ showed an *m/z* 708.3010 in MS1 ([C_38_H_45_NO_12_ + H]^+^, exact mass *m/z* 708.3015, Δ*m/z* = 0.64 ppm), and an abundant fragment of *m/z* 182.0818 in MS/MS which may originate from the cleavage of the remaining phenylpropionic acid side chain and the taxane nucleus ([C_9_H_11_NO_3_ + H]^+^, exact mass *m/z* 182.0812, Δ*m/z* = −3.46 ppm). ‘Epi-carbamate’ showed a similar product ion spectrum with the same base peak of *m/z* 182.0820 ([C_9_H_11_NO_3_ + H]^+^, exact mass *m/z* 182.0812, Δ*m/z* = −4.56 ppm) and *m/z* 708.3004 ([C_38_H_45_NO_12_ + H]^+^, exact mass *m/z* 708.3015, Δ*m/z* = 1.47 ppm) in MS1.

An exemplary chromatogram of the degradation products following two applications of 70 µg/mL docetaxel for 48 h each is shown in Figure S2. We found only trace amounts of docetaxel degradation products in the TOM model media following the two applications of 7 µg/mL docetaxel for 48 h each. Therefore, we did not consider the degradation products in the real-time pharmacokinetic analyses.

### 3.3. Validation

As method A is used for quantitation in the online hyphenation of the tumor model with UHPLC based analysis, it was validated according to the guideline of the EMA [15].

Selectivity and carry-over: The method fulfilled the criteria for selectivity (<20% response in blank artificial matrix compared to response obtained at LLOQ) with a maximum of 7.45% detector response. Carry-over was a more critical parameter since the concentration range was very broad. Even after the optimization of the injector wash procedures, the detector response of analyte-free matrix samples exceeded the allowed 20% LLOQ detector response with a maximum of 29.42% after injection of HQC samples. Therefore, additional blank sample injections were included after samples of high concentrations resulted in successful prevention of carry-over.

Lower limit of quantitation and calibration: The concentration of 0.001 µg/mL showed acceptable accuracy (92.42–114.17%) and precision (6.58%CV) and was therefore chosen as the lowest point of the calibration. Based on the EMA guideline, the calculated LLOQ was 0.16 ng/mL and LOD 0.05 ng/mL, respectively.

For the calibration function, a quadratic fit after log-log transformation of the data provided the best results in terms of a combination of low residuals and best overall accuracy. All CAL samples met the requirements by EMA.

Accuracy and precision: The method (A) fulfilled the requirements given by EMA. Calculated concentrations of QC and CAL samples were within ±15% of the nominal values (Table 3), only 8.33% (within-day) and 15% (between-day) with only individual values outside.

### 3.4. Docetaxel Pharmacokinetics in TOM Models

The area under the concentration curves (AUC), the maximum concentration (C_max_), and the time to maximum concentration (t_max_) as main pharmacokinetic parameters for the concentration versus time profiles of docetaxel within the sampling port are summarized in Table 4.

**Figure 3:**
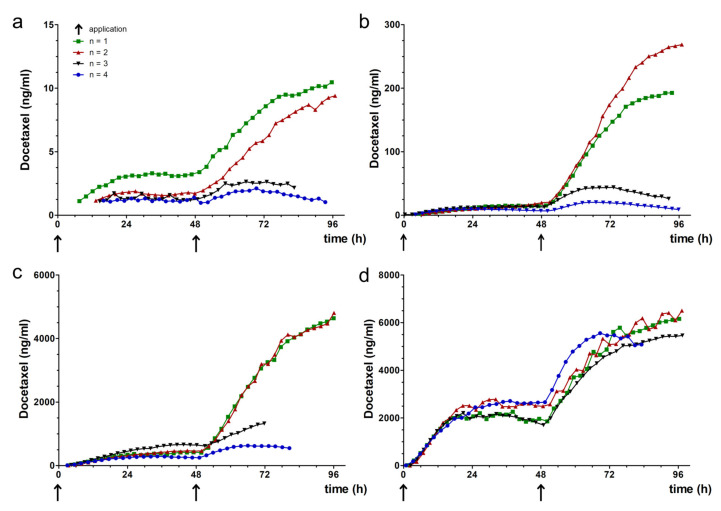
Concentration-time curves of docetaxel. Docetaxel concentrations in the sampling port of TOM models following the application of (**a**) 7, (**b**) 70, (**c**) 700 or (**d**) 7000 ng/mL docetaxel. Docetaxel was supplemented to the differentiation at 0 and 48 h (arrows), *n* = 4 for each concentration.

The course of the concentration versus time curves was comparable between the applied drug doses (Figure 3). Following the administration of docetaxel by supplementing the differentiation medium of TOM models in the reservoir at 0 h, the drug concentration increased until a plateau phase. The time to maximum concentration t_max_, 39 ± 7.9 h was almost independent of the administered docetaxel dose, while the C_max_ depended on the administered docetaxel dose. Following the exchange of the differentiation medium, again supplemented with the same docetaxel doses, we detected 2.4- to 9.1-fold increased maximum concentrations and 2.4- to 8.8-fold increased AUCs in the sampling port compared to the respective values following the first docetaxel administration. Furthermore, we detected about 4- to 7-fold higher docetaxel concentrations in the sampling port compared to the applied docetaxel concentration (Figure 3b,c). This effect was not observed when applying 7 or 7000 ng/mL docetaxel (Figure 3a,d). Again, the t_max_ values were close to the end of the treatment cycle with values ranging between 82 and 89 h.

Moreover, the concentration versus time curves showed a different shape in two experiments (blue and black curve vs. red and green curve in Figure 3a–c). The slope of the blue and black curves markedly differed from the slope of the red and green curves after the second docetaxel administration. The relatively constant docetaxel concentrations within the sampling port could result from evaporation of medium from the reservoir (Figure 1a(3)), causing in loss of contact of the model with the reservoir. Evaporation also affected the accessibility of the sample fluid for the autosampler needle, since we did not measure docetaxel from certain time points on (e.g., black curve in Figure 3c).

## 4. Discussion

An automated UHPLC-MS/MS method with online sampling in TOM models is presented here. This proof-of-concept study demonstrated the feasibility of real-time monitoring of drug levels in TOM models without any sample preparation. To achieve this, both the analytical method for docetaxel quantitation in human blood samples [20] and the culture of TOM models [14] needed to be adapted only slightly. Our approach was validated according to EMA guidelines [15] and is easily transferrable to other in vitro disease models.

In vitro studies frequently use drug doses far higher than the maximum tolerated dose in patients [21]. This overdosing causes effects in vitro that are not reproducible in vivo, contributing to the high attrition rate of investigational new drugs in clinical trials. Even if the patients tolerate such high doses, they will be prone to off-target effects. Aside from the bench-to-bedside extrapolation of drug doses, clinical data can be useful to conduct more relevant studies for investigation of personalized adaptations. Considering the maximum plasma concentration at the highest single dose recommended in the drug product of marketed drugs provides an upper limit for in vitro studies [22]. This concept is particularly useful to test potential new indications for approved drugs. We used docetaxel as a model drug to develop our analytical approach since both the efficacy and the pharmacokinetics of docetaxel are well-known [22]. After calculating a steady-state concentration of 74 ng/mL docetaxel in patients following an intravenous application of 75 mg/m^2^ (for details, see [14]), we selected 7; 70; 700; and 7000 ng/mL as test concentrations in TOM models. The AUC within the TOM models ranged between 66.32 and 85,658.15 h × ng/mL following the first, and between 151 and 211,171 h×ng/mL following the second docetaxel application. Together with C_max_ values below 2492 and 5920 ng/mL, these in vitro results were in range of the clinical application of 100 mg/m^2^, which results in an AUC of 4600 h × ng/mL and C_max_ of 3700 ng/mL [23].

Focusing on the nominal concentration of 70 ng/mL, we detected less docetaxel in the sampling port than has been found in human blood samples. This discrepancy supports the hypothesis of the poor uptake of anticancer drugs into solid tumors [6]. Likewise, paclitaxel penetrates only to the periphery of spheroids [5]. Nevertheless, docetaxel uptake into the TOM models increased following the second drug application. Since apoptosis results in enhanced drug uptake into inner cell layers of solid tumors [8], tumor cells dying after the first application should favor docetaxel uptake into TOM models.

Moreover, our method provides an in-depth insight into the formation of docetaxel degradation products. Since docetaxel epimerization is associated with a loss of potency and tumor resistance development in vivo [24], the considerable epimer formation will affect the efficacy of docetaxel. In contrast, the trace amounts of oxidation products and carbamates should not limit docetaxel effects in TOM models, although they are 10- to 40-fold less active [25]. The degradation products were identified by QTOF-MS and related to degradation products known from the literature [26]. Nevertheless, our approach allows for only limited insights into clinically relevant clearance due to the absence of hepatic metabolism and biliary excretion. If tumor cells metabolize the applied drugs, the quantitation of local metabolites will be feasible as well, but in the case at hand, we observed docetaxel epimerization and formation of degradation products as artifacts also in cell-free medium.

Differences between docetaxel concentrations in human patients and TOM models also arise from differences in protein binding. Whereas plasma protein binding of docetaxel is 97% in the patients [22], protein binding in medium containing fetal bovine serum is saturable. Paclitaxel, close in chemical structure to docetaxel, shows a protein binding between 79% at 500 ng/mL and 20% at 15,000 ng/mL [27]. Thus, we expect higher amounts of free drug available compared to the patients, especially following the application of 7000 ng/mL docetaxel. Nevertheless, we were not able to discriminate free against total docetaxel concentration, since the membrane of the sampling port has a pore size of 400 nm. Most protein sizes range between 1 and 100 nm, making protein diffusion into the sampling port likely. This might also explain higher C_max_ values in the sampling port than the administered concentration in the reservoir, since we quantified all docetaxel within the sampling port. The first docetaxel administration saturated the protein binding and intracellular fluids, the second application directly increased the concentration in the interstitial fluid of TOM models and the sampling port. However, we assume complete equilibration between the interstitial fluid of the TOM model and the sampling fluid within two hours, equal to the time interval we selected between two measurements. Thus, signals of the concentration over time curve earlier between zero and two hours might not recapitulate the concentration within the interstitial fluid of the TOM model, but provide an insight into the lag-time between docetaxel application and first appearance within the sampling port. As to be expected, the lag-time decreases with increasing docetaxel concentrations: 11.1 ± 3 h (7 ng/mL docetaxel application) compared to 1.82 ± 0.6 h (7000 ng/mL docetaxel application).

Since classical microdialysis already allowed insights into tissue-specific drug [28] and cytokine levels [29], the automated determination of pharmacokinetic profiles will enable patient-specific analyses in higher throughput. PK-PD modelling already improved dose selection and characterization of drug effects on tumor growth, overall survival and safety [30], but requires relevant data for the patient and his/her tumor. Nonclinical testing together with pharmacometrics may provide a more detailed insight by testing drugs in patient-specific models and extrapolating drug concentrations in tumors to adapt dose regimen for patients.

UHPLC-MS/MS again proved as the method of choice as it was already useful for a wide range of applications in pharmacology, toxicology, and forensics [31,32,33]. Despite first dilute and inject attempts to reduce the time-consuming sample preparation [34,35,36,37,38,39], UHPLC-MS/MS analyses still often utilizes extensive sample preparation to separate the molecule of interest from interfering proteins and potential enzymatic degradation processes [40]. Our method (A) used for quantitation of docetaxel was successfully validated in terms of selectivity, carry-over, lower limit of quantitation (LLOQ), calibration function, accuracy, and precision according to EMA guidelines for bioanalytical method validation. A very broad concentration range of 1–10,000 ng/mL was covered compared to already published methods [20,41], allowing the analysis of docetaxel administered ranging from 7 to 7000 ng/mL. The method proved to be accurate and precise, showed acceptable carry-over after including blank injections between high and low concentration samples, as well as fitness-for purpose in LLOQ. Furthermore, the method was fast, being able to separate docetaxel and 7-epi-docetaxel in less than 3.7 min (total run-time including cleaning of injector 9.75 min).

Future studies will compare differences between the patients’ drug responses and drug delivery systems to optimize the dose regimen and application form. For increased efficacy, model size and sampling volume may be further optimized in the direction of high-throughput, and therefore, enhance personalized medicine.

## 5. Conclusions

We developed and evaluated a real-time approach to automatically measure docetaxel concentrations in TOM models. Partial epimerization and neglectable amounts of degradation products were detected instantaneously upon application of docetaxel to the medium. The courses of concentration versus time curves for 96 h were comparable among four different docetaxel concentrations. The first drug application resulted in an increase of docetaxel concentration, followed by a plateau phase, and exceeded after the second drug application. This proof-of-concept study paves the way for real-time pharmacokinetic and further online investigations in 3D tumor models and beyond, and thus, helps to improve preclinical drug development and personalized medicine.

## Figures and Tables

**Table 1:** Multiple reaction monitoring (MRM) transitions of docetaxel sodium adduct, used in method A.

Precursor Ion (*m/z*)	Product Ion (*m/z*)	Collision Energy	Cell Accelerator Voltage	Polarity
830.3	549.1	25	4	Positive
830.3	304.1	20	2	Positive

**Table 2:** LC-MS data of docetaxel (highlighted gray) and postulated degradation products, acquired using method B.

Degradation Product	Formula	RT (min)	*m/z*	Exact Mass	Adduct	Mass Accuracy (ppm)
**Carbamate**	C_38_H_45_NO_12_	4.30	708.3010	708.3015	[M + H]^+^	0.65
**10DABIII**	C_29_H_36_O_10_	4.55	545.2378	545.2381	[M + H]^+^	0.60
**Epi-carbamate**	C_38_H_45_NO_12_	4.72	708.3004	708.3015	[M + H]^+^	1.47
**Epi-10DABIII**	C_29_H_36_O_10_	5.31	567.2196	567.2201	[M + Na]^+^	0.76
**Oxo-10DABIII**	C_29_H_34_O_10_	5.40	565.2041	565.2044	[M + Na]^+^	0.56
**Epi-oxo-10DABIII**	C_29_H_34_O_10_	5.84	565.2040	565.2044	[M + Na]^+^	0.67
**Docetaxel**	C_43_H_53_NO_14_	7.95	830.3374	830.3358	[M + Na]^+^	−1.9
**Epi-Docetaxel**	C_43_H_53_NO_14_	9.08	830.3377	830.3358	[M + Na]^+^	−2.26
**Oxo-Docetaxel**	C_43_H_51_NO_14_	9.94	828.3200	828.3202	[M + Na]^+^	0.19
**Epi-oxo-Docetaxel**	C_43_H_51_NO_14_	11.07	828.3192	828.3202	[M + Na]^+^	1.16

**Table 3:** Accuracy and precision. c: docetaxel concentration, CV: coefficient of variation, RE: Relative error as measure of accuracy, LLOQ: lower limit of quantitation, LQC: lower quality control, MQC: middle quality control, HQC: higher quality control.

QC		Within-day (*n* = 5)			Between-day (*n* = 5)		
	Expected c (ng/mL)	Mean Calculated c (ng/mL)	CV (%)	RE (%)	Mean Calculated c (ng/mL)	CV (%)	RE (%)
**LLOQ**	1.00	1.06	7.26	5.71	1.01	11.40	1.62
**LQC**	3.00	2.76	2.27	−7.87	2.67	3.37	−10.94
**MQC**	3000	3027	7.83	0.89	3254	9.48	8.48
**HQC**	7500	6982	7.79	−6.91	7676	9.57	2.35

**Table 4:** Main pharmacokinetic parameters following 1st docetaxel application (0–48 h) and 2nd docetaxel application (48–96 h). c: docetaxel concentration, AUC: area under the curve.

**c**	**AUC (0–48 h)**		**C_max_ (0–48 h)**		**t_max_ (0–48 h)**	
**(ng/mL)**	**Mean** **(h × ng/mL)**	**CV** **(%)**	**Mean** **(ng/mL)**	**CV** **(%)**	**t_max_** **(h)**	**CV** **(%)**
7	66.3	44.6	1.9	194.5	43.9	14.5
70	444.4	12.5	14.4	25.3	39.5	20.7
700	13,324	26.0	461	29.0	41	9.9
7000	85,658	8.3	2492	10.4	32	21.4
						
**c**	**AUC (48–96 h)**		**C_max_ (48–96 h)**		**t_max_ (48–96 h)**	
**(ng/mL)**	**Mean** **(h × ng/mL)**	**CV** **(%)**	**Mean** **(ng/mL)**	**CV** **(%)**	**t_max_** **(h)**	**CV** **(%)**
7	151.4	84.4	6.0	65.1	82.8	16.2
70	3915.1	75.0	131.4	78.5	82.7	15.5
700	78,890	75.1	2850	66.3	83	16.6
7000	211,171	12.2	5920	7.3	90	13.7

## Supplementary Materials

The following are available online at https://www.mdpi.com/1999-4923/12/5/413/s1, Figure S1: Multiple reaction monitoring (MRM) chromatogram, Figure S2: Overlay of extracted ion chromatograms of docetaxel and degradation products, Figure S3: Product ion spectra of degradation products, Figure S4: Suggested chemical structure of docetaxel and degradation products, structural differences in comparison to docetaxel are displayed in red color.
