# Supplementary material for: Automated Real-Time Tumor Pharmacokinetic Profiling in 3D Models: A Novel Approach for Personalized Medicine
Source: Pharmaceutics. 2020 Apr 30;12(5):413. doi: 10.3390/pharmaceutics12050413 (PMC7284432; doi:10.3390/pharmaceutics12050413)
Supplement: Supplementary file 1 [file pharmaceutics-12-00413-s001.pdf]

pharmaceutics

Supplementary Materials: Automated Real-Time Tumor Pharmacokinetic Profiling in 3D Models: A Novel Approach for Personalized Medicine

Jan F. Joseph, Leonie Gronbach, Jill García-Miller, Leticia M. Cruz, Bernhard Wuest, Ulrich Keilholz, Christian Zoschke and Maria K. Parr

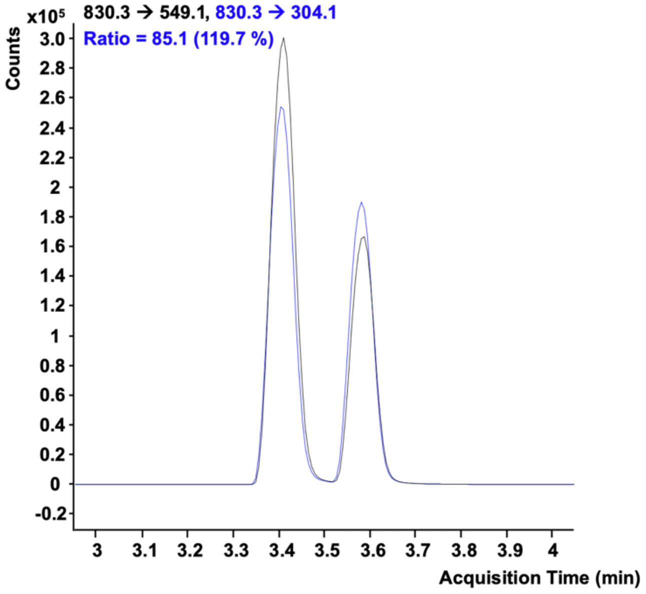

Figure S1. Multiple reaction monitoring (MRM) chromatogram. Product ions of docetaxel (left peak) and epi-docetaxel (right peak) acquired using method A.

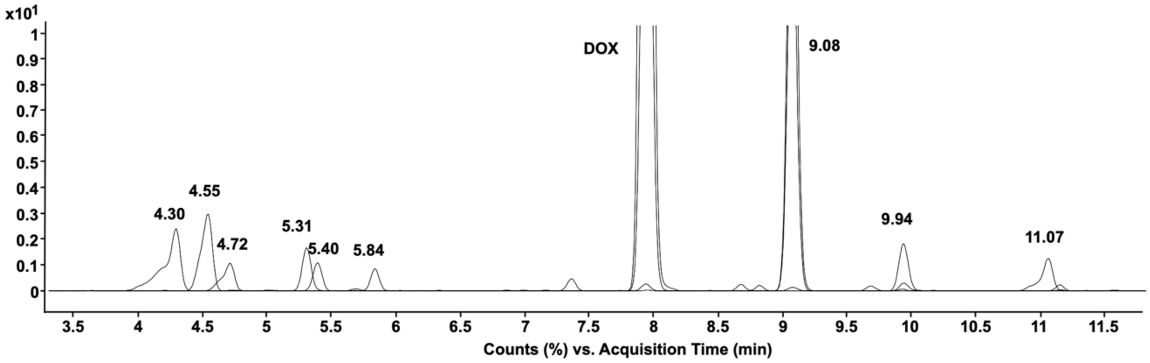

Figure S2. Overlay of extracted ion chromatograms of docetaxel and degradation products, acquired using method B.

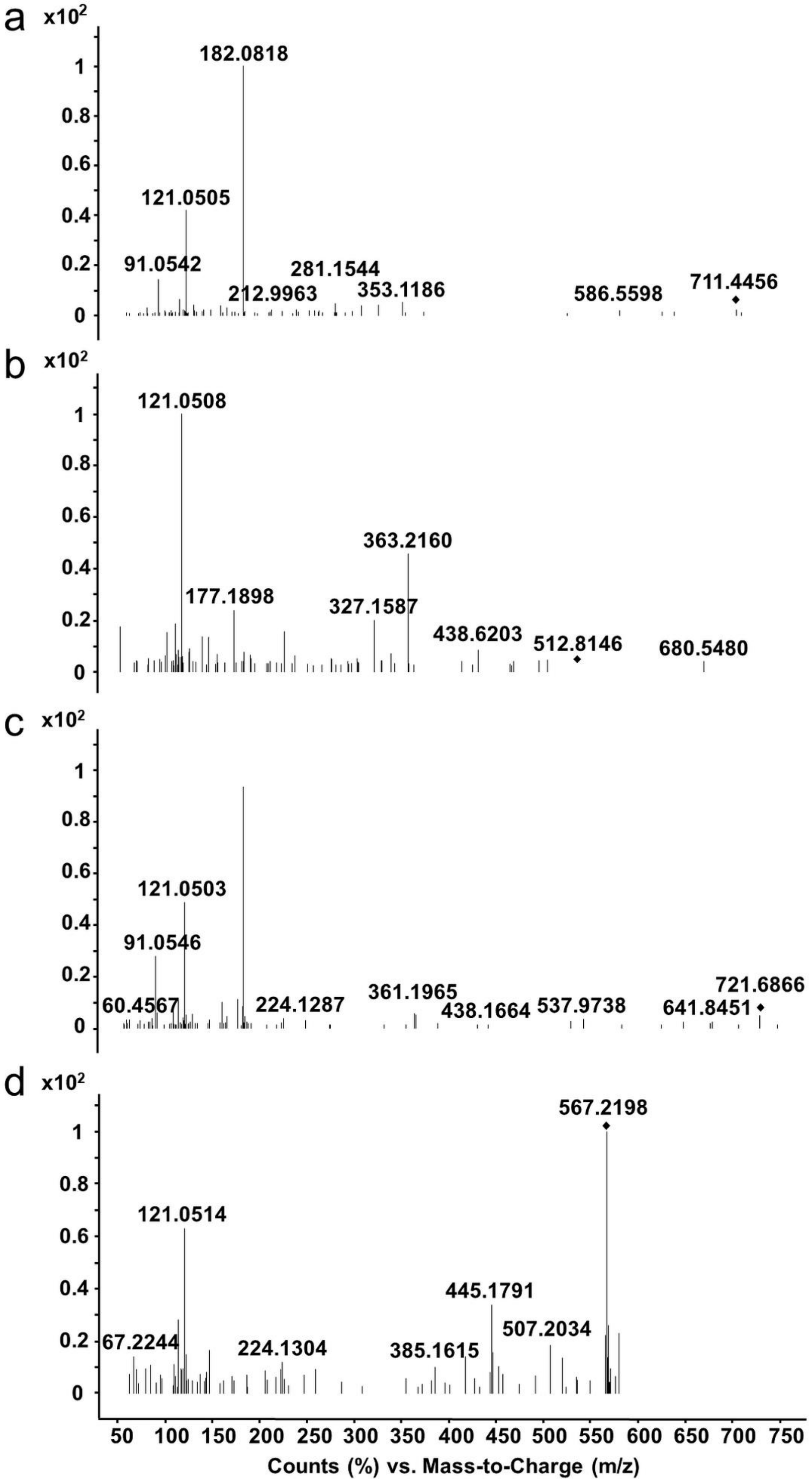

Figure S3. Product ion spectra of degradation products acquired using method B. (**a**) Carbamate (RT: 4.30 min, precursor $[M+H]^+$=708.3010), (**b**) 10-deacetyl baccatin III (RT: 4.55 min, precursor $[M+H]^+$=545.2378), (**c**) epi-carbamate (RT: 4.72 min, precursor $[M+H]^+$=708.3004), (**d**) 7-epi-10-deacetyl baccatin (RT: 5.31 min, precursor $[M+Na]^+$=567.2196).

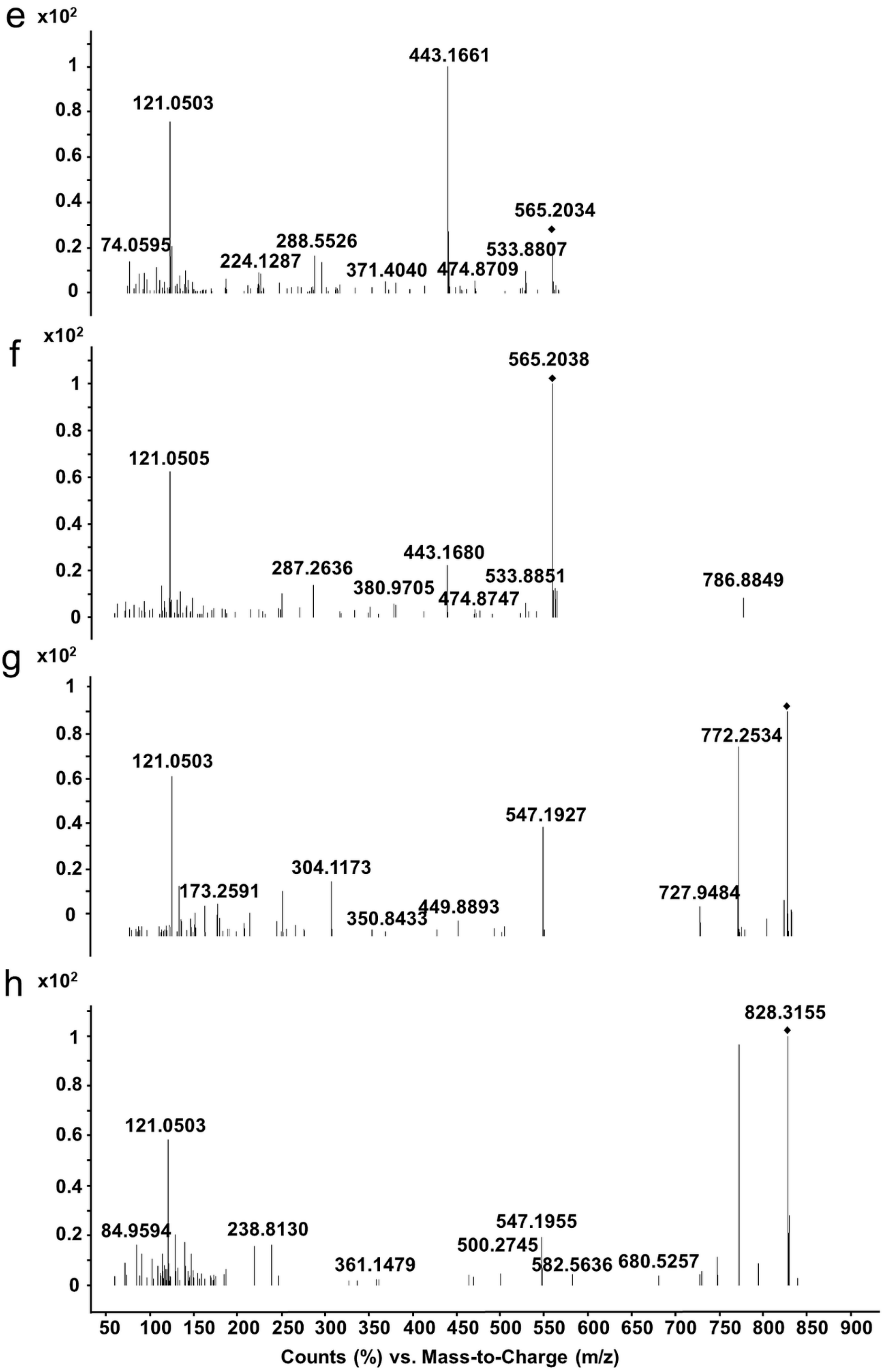

Figure S3. (continued). Product ion spectra of degradation products acquired using method B. (**e**) 10-oxo-10-deacetyl baccatin III (RT: 5.40 min, precursor $[M+Na]^+$=565.2041), (**f**) 7-epi-10-oxo-10-deacetyl baccatin III (RT: 5.84 min, precursor $[M+Na]^+$=565.2040), (**g**) 10-oxo-docetaxel (RT: 9.94 min, precursor $[M+Na]^+$=828.3200), (**h**) 7-epi-10-oxo-docetaxel (RT: 11.07 min, precursor $[M+Na]^+$=828.3192).

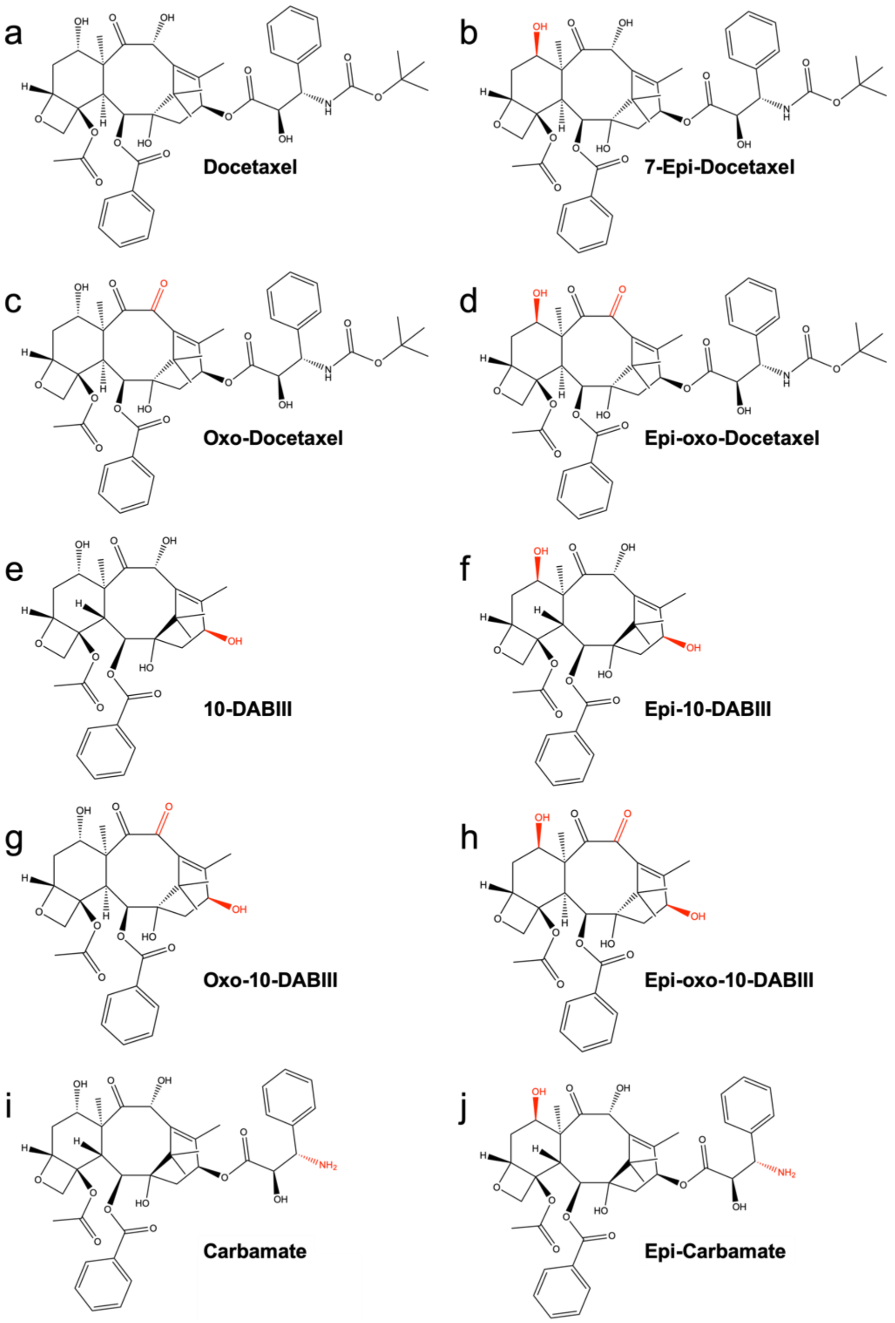

Figure S4. Suggested chemical structure of docetaxel and degradation products, structural differences in comparison to docetaxel are displayed in red color.
